# Educational inequalities in hypertension: complex patterns in intersections with gender and race in Brazil

**DOI:** 10.1186/s12939-016-0441-6

**Published:** 2016-11-17

**Authors:** Ronaldo Fernandes Santos Alves, Eduardo Faerstein

**Affiliations:** Department of Epidemiology, Institute of Social Medicine, State University of Rio de Janeiro, Rua São Francisco Xavier 524, 7° andar, blocos D e E, Maracanã, Rio de Janeiro, RJ 20550-013 Brazil

**Keywords:** Health Status Disparities, Educational status, Gender Identity, Ethnic Groups, Hypertension, Cross-Sectional Studies, Intersectionality

## Abstract

**Background:**

Hypertension is a major public health issue worldwide, but knowledge is scarce about its patterns and its relationship to multiple axes of social disadvantages in Latin American countries. This study describes the educational inequality in the prevalence of hypertension in Brazil, including a joint stratification by gender and race.

**Methods:**

We analyzed interview-based data and blood pressure measurements from 59,402 participants aged 18 years or older at the 2013 Brazilian National Health Survey (PNS). Sociodemographic characteristics analyzed were gender (male, female), racial self-identification (white, brown, black), age (5-years intervals), and educational attainment (pre-primary, primary, secondary, tertiary). Hypertension was defined as systolic blood pressure ≥ 140 mmHg and/or diastolic blood pressure ≥ 90 mmHg, and/or self-reported use of antihypertensive medications in the last 2 weeks. We used logistic regression to evaluate the age-adjusted prevalences of hypertension (via marginal modeling), and pair-wise associations between education level and odds of hypertension. Further, the educational inequality in hypertension was summarized through the Relative Index of Inequality (RII) and the Slope Index of Inequality (SII). All analyses considered the appropriate sampling weights and intersections with gender, race, and education.

**Results:**

Age-adjusted prevalence of hypertension was 34.0 % and 30.8 % among men and women, respectively. Black and brown women had a higher prevalence than whites (34.5 % vs. 31.8 % vs. 29.5 %), whereas no racial differences were observed among men. White and brown, but not black women, showed graded inverse associations between hypertension and educational attainment; among men, non-statistically significant associations were observed in all racial strata. The RII and SII estimated inverse gradients among white (RII = 2.5, SII = 18.1 %) and brown women (RII = 2.3, SII = 14.5 %), and homogeneous distributions of hypertension in educational subgroups among black women and among men.

**Conclusion:**

In this representative sample of Brazilian adults, the association between educational attainment and hypertension was influenced by gender and race – a topic still poorly understood. Our findings highlight the importance of assessing intersections of multiple sociodemographic characteristics in health inequalities research. The use of comprehensive measures of inequality, such as RII and SII, provide useful insights for monitoring health inequalities in an intersectional perspective.

## Background

The primary causes of death worldwide are cardiovascular diseases, and more than half of these deaths are due to complications of hypertension [1, 2]. Most individuals with hypertension live in low- and middle-income countries, where its degrees of awareness, treatment and control tend to be lower compared to high-income countries [1]. Moreover, the prevalence of hypertension in the Latin America and Caribbean populations is the highest among developing countries [3].

In Brazil, an upward trend in the prevalence of self-reported hypertension was observed since 1998 [4], reaching 21.4 % of the adult population in 2013 [5]. During this period, the prevalence was persistently higher among women and persons of low socioeconomic position [4–6]. Besides, surveys in the city of Rio de Janeiro documented higher frequency of hypertension among blacks of both genders [7], as well as a steeper educational gradient in hypertension among blacks exposed to racial discrimination than blacks with no history of perceived racism or whites [8]. In addition, women showed a stronger inverse association between socioeconomic position and hypertension than males in countries such as Trinidad and Tobago, Austria and Norway [9–11], whereas the magnitude and even the direction of social inequalities in its occurrence varied, for example, in Argentina and India [12, 13].

Overall, a variety of sociodemographic attributes, most prominently low education, male gender, and dark-skinned color, have been associated with hypertension [3, 14, 15]. However, epidemiologic evidence is scarce regarding the patterns of hypertension in emerging economies and about its relationship to multiple axes of social disadvantages. The present study intended to describe educational inequalities related to the prevalence of hypertension in intersections with gender and race in Brazil. Of note, we used both the Relative Index of Inequality (RII) and the Slope Index of Inequality (SII) which offer summary estimates useful for the purpose of health equity monitoring, and have the potential to advance intersectional population health research.

## Methods

### Setting and study design

A cross-sectional study was carried out with data obtained from the 2013 Brazilian National Health Survey (PNS), a nationwide household survey conducted by the Brazilian Institute of Geography and Statistics (IBGE) in partnership with the Brazilian Ministry of Health. The PNS is part of the IBGE Integrated System for Household Surveys, and it is mainly focused on the production of information about the health status and lifestyles of the Brazilian population, and also about the access and use of health services, preventive actions, continuity of care, and health care financing.

Detailed information on the PNS methodology is available elsewhere [16, 17]. In summary, the sampling design involved a three-stage cluster sampling with stratification of the primary units of sampling (PSU). The census tracts or a set of sectors of the 2010 Geographic Operating Base formed the PSU; the households were the second-stage units, and the residents aged 18 years or older were the third-stage units. One adult among all the eligible residents was selected with equal probability both to answer to the individual questionnaire and to measure casual blood pressure. Interviews and measurements were scheduled according to the availability of the adults selected, and they were performed by trained personnel from the IBGE and documented with the assistance of handheld computers – PDA (personal digital assistant). The PDA included a built-in component of consistency checks. The data collection occurred from August 2013 to February 2014 and obtained 64,348 household interviews (proportion of loss of 20.8 %) with 60,202 individuals’ surveyed (proportion of non-response of 8.1 %), and 59,402 people with their blood pressure measured (proportion of missing data of 1.0 %).

Sample weights were defined for the PSU, for households and all their residents, in addition to the weight for the selected resident. This last one was calculated considering (a) the weight of the related household, (b) the resident selection probability, (c) adjustments of non-response by sex and (d) calibration of population totals by sex and age groups, estimated with the weight of all residents combined.

### Measures

A multidimensional structured questionnaire was used to collect information in the PNS. We analyzed the open data from IBGE (current version, 2016/06/30) [18] regarding the following sociodemographic variables: gender (male, female), age in years (18 to 24, 25 to 29, 30 to 34, 35 to 39, 40 to 44, 45 to 49, 50 to 54, 55 to 59, 60 to 64, 65 to 69, 70 to 74, 75 to 79, 80 or older), and race (white, brown, black, ‘other’). In IBGE classification, brown is a cognate term for *pardo*, which is a broad classification that encompasses mixed-race Brazilians. The ‘other’ group includes Asian and indigenous individuals. Because of the heterogeneity and small sample size of the ‘other’ group, we considered only whites, browns, and blacks in our main analysis.

Education was selected as our indicator of socioeconomic position. We aggregated the seven categories of education on four principal levels, considering the International Standard Classification of Education [19]: pre-primary (no schooling and incomplete primary school); primary (complete primary school and incomplete secondary school); secondary (complete secondary school and incomplete tertiary school), and tertiary (complete tertiary school or more).

Hypertension was defined as systolic blood pressure equal to or higher than 140 mmHg, and/or diastolic blood pressure equal to or higher than 90 mmHg, and/or self-reported use of antihypertensive medications in the last 2 weeks. Blood pressure was measured three times using a calibrated digital device, with two-minute intervals between them, after individuals’ resting for at least five minutes; analyses were based on the mean of the two last readings. The blood pressure measurements followed recommendations of the Seventh Report of the Joint National Committee on Prevention, Detection, Evaluation, and Treatment of High Blood Pressure [20]. In addition, self-reported use of antihypertensive medications was measured using the following question: During the last two weeks, have you taken medications because of hypertension (high blood pressure)?

### Statistical analysis

Descriptive analyses included frequencies of the sociodemographic characteristics and of the outcome of interest. Prevalence of hypertension was age-adjusted using 5-year intervals, marginal modeling and interactions with the sociodemographic factors [21]. The standard population was the total PNS population (men and women).

Odds ratios (OR) and 95 % confidence intervals (95 % CI) for the occurrence of hypertension were estimated by using logistic regression models for those with pre-primary, primary, and secondary education as compared to those reporting tertiary level of education. The Relative Index of Inequality (RII) and the Slope Index of Inequality (SII) [22–24] were used to estimate the magnitude and direction of educational inequalities in the occurrence of hypertension. The RII (logit link) and SII (identity link) are also based on regression models, but the independent variable (exposure) is defined from the cumulative relative frequency of the study population according to education levels. Differently from the OR, which assigns ordinal values to individuals from the respective educational levels, the RII and SII attribute numerical scores which consider the population size related to the various categories of education, as described by Alves e Faerstein (2015) [25]:

$$ \mathrm{Score}\ \mathrm{k}=\left[\left({\varSigma}_{i=1}^{k-1}{f}_i+{f}_k/2\right) \div N\right] $$

Where *k* is the (ordinal) index of the education strata; *f*_*k*_ is the *k*’s group absolute frequency, and N is the total of individuals in the population. The ordering started from the most educated and the numeric score was calculated separately for each sociodemographic subgroup. Age was included as a discrete variable (years) in the logistic regression models.

All estimates were based on the complex sample of adults aged 18 years or older, considering the appropriate sampling weights. The analyses were stratified by gender and race, and they were processed in R 3.3.1 [26]. The “survey” library [27, 28] was used to correct the sampling plan design effect.

## Results

Table 1 presents the sociodemographic characteristics of the study population and the prevalence of hypertension in the sociodemographic strata. There was a higher proportion of women than men, 51 % of Brazilian adults self-identified as brown or black, and half of the population aged between 25 and 49 years old. On the education, 39 % did not have formal education diploma and 45 % attained at least secondary level.

**Table 1:** Sociodemographic characteristics and prevalence of hypertension (*N* = 59,402). National Health Survey, Brazil, 2013

Variable	Sample (*N*)	Population (%)^b^	% Hypertension (95 % CI)^b^
Brazil	59402	100.0	32.3 (31.6–33.1)
Gender			
Men	25920	47.6	33.0 (32.0–34.1)
Women	33482	52.4	31.7 (30.8–32.6)
Age group (years)			
18–24	7542	15.7	6.4 (5.3–7.5)
25–29	6280	10.0	11.1 (9.6–12.5)
30–34	7242	11.3	16.5 (14.9–18.1)
35–39	6761	10.2	21.4 (19.8–23.1)
40–44	5945	9.1	29.1 (27.1–31.1)
45–49	5425	9.1	37.0 (34.8–39.2)
50–54	4814	8.6	45.9 (43.3–48.4)
55–59	4216	7.8	53.6 (51.0–56.3)
60–64	3465	5.8	58.8 (55.9–61.7)
65–69	2773	4.5	65.4 (62.6–68.2)
70–74	2052	3.3	70.5 (67.0–73.9)
75–79	1389	2.1	73.1 (69.0–77.1)
80 or older	1498	2.5	72.2 (68.7–75.8)
Race/Skin color			
White	23828	47.5	33.4 (32.3–34.5)
Brown	29066	41.9	30.2 (29.2–31.2)
Black	5568	9.2	36.5 (34.4–38.6)
Other^a^	940	1.4	30.0 (24.6–35.5)
Education			
Pre–primary	23882	39.1	45.1 (43.9–46.2)
Primary	9061	15.5	26.1 (24.4–27.7)
Secondary	18807	32.7	22.2 (21.1–23.4)
Tertiary	7652	12.7	26.5 (24.7–28.4)

*CI* confidence interval

^a^The ‘other’ group includes Asian and indigenous individuals; because of the heterogeneity and small sample size of the ‘other’ group, we included only whites, browns, and blacks in our further analyses

^b^The estimates are based on the sample of 59,402 adults aged 18 years or older, considering the appropriate sampling weights

^b^Hypertension was defined as systolic blood pressure ≥ 140 mmHg and/or diastolic blood pressure ≥ 90 mmHg, and/or self-reported use of antihypertensive medications in the last 2 weeks (proportion of missing data of 1.0 %)

Overall, the PNS prevalence of hypertension was 32.3 %. This prevalence increased with age in both genders (*p* < 0.001), and it was slightly higher among men as compared to women. There was a higher age-adjusted prevalence among black (*p* < 0.001), compared with white people (data not shown). The prevalence of hypertension was significantly higher among those without primary education, compared to those with university level.

Table 2 presents the sociodemographic characteristics and the prevalence of hypertension considering joint stratification by education, gender, and race. Women showed higher educational levels: 13.9 % of them had a university degree, whereas among men, 11.4 % had it. Additionally, 2.3 to 3.2 times more whites than browns or blacks reported university degrees in both genders.

**Table 2:** Educational inequalities in hypertension according to gender and race. National Health Survey, Brazil, 2013

Variable	% Population	% Hypertension (95 % CI)	Age-adjusted % Hypertension (95 % CI)^b^	Unadjusted OR (95 % CI)	Age-adjusted OR (95 % CI)^a^
All Men					
Tertiary	11.4	33.7 (30.7–36.7)	33.2 (30.8–35.6)	1.0	1.0
Secondary	32.2	25.0 (23.1–26.8)	34.7 (32.7–36.7)	0.7 (0.6–0.8)	1.0 (0.8–1.2)
Primary	16.5	26.0 (23.5–28.4)	33.5 (30.9–36.1)	0.7 (0.6–0.8)	1.0 (0.8–1.2)
Pre-primary	39.8	42.2 (40.6–43.9)	33.9 (32.5–35.4)	1.4 (1.2–1.7)	1.1 (0.9–1.2)
Total	100.0	33.0 (32.0–34.1)	34.0 (33.0–35.0)	-	-
RII^a^	-	2.7 (2.3–3.3)	1.1 (0.9–1.3)	-	-
SII (%)^a^	-	21.8 (17.9–25.8)	2.2 (−1.3–5.7)	-	-
White Men					
Tertiary	16.9	34.4 (30.6–38.3)	32.1 (29.1–35.1)	1.0	1.0
Secondary	34.7	26.9 (24.2–29.7)	35.1 (32.1–38.0)	0.7 (0.6–0.9)	1.1 (0.9–1.3)
Primary	15.3	26.0 (22.4–29.7)	31.1 (27.3–34.9)	0.7 (0.5–0.9)	0.9 (0.7–1.2)
Pre-primary	33.1	47.7 (45.0–50.4)	35.3 (33.1–37.5)	1.7 (1.4–2.1)	1.2 (1.0–1.5)
Total	100.0	34.9 (33.3–36.6)	34.0 (32.6–35.5)	-	-
RII^a^	-	2.8 (2.1–3.7)	1.2 (0.9–1.6)	-	-
SII (%)^a^	-	23.1 (17.3–28.9)	4.7 (−0.4–9.8)	-	-
Brown Men					
Tertiary	6.5	29.6 (24.5–34.7)	34.6 (30.2–39.1)	1.0	1.0
Secondary	30.2	22.5 (19.9–25.2)	34.0 (31.1–37.0)	0.7 (0.5–0.9)	0.9 (0.7–1.2)
Primary	17.2	24.2 (20.9–27.5)	35.0 (31.4–38.7)	0.8 (0.6–1.0)	1.0 (0.7–1.3)
Pre-primary	46.2	37.4 (35.1–39.7)	31.9 (30.0–33.9)	1.4 (1.1–1.9)	0.9 (0.7–1.2)
Total	100.0	30.2 (28.6–31.7)	33.2 (31.7–34.7)	-	-
RII^a^	-	3.0 (2.3–3.9)	0.9 (0.7–1.3)	-	-
SII (%)^a^	-	22.4 (17.0–27.8)	−0.3 (−5.3–4.7)	-	-
Black Men					
Tertiary	5.3	27.7 (13.8–41.6)	33.5 (18.9–48.0)	1.0	1.0
Secondary	28.5	26.2 (20.8–31.6)	36.0 (30.7–41.3)	0.9 (0.4–2.0)	1.1 (0.5–2.5)
Primary	20.2	33.3 (23.5–43.2)	37.7 (28.3–47.2)	1.3 (0.6–3.0)	1.3 (0.5–3.1)
Pre-primary	46.0	44.7 (39.6–49.9)	37.6 (33.2–41.9)	2.1 (1.0–4.4)	1.3 (0.6–2.9)
Total	100.0	36.2 (32.7–39.8)	37.0 (33.9–40.2)	-	-
RII^a^	-	3.8 (2.2–6.7)	1.4 (0.7–2.5)	-	-
SII (%)^a^	-	30.0 (17.8–42.2)	6.8 (−5.0–18.6)	-	-
All Women					
Tertiary	13.9	21.2 (19.0–23.4)	22.9 (20.8–25.0)	1.0	1.0
Secondary	33.0	19.8 (18.4–21.2)	27.3 (25.8–28.9)	0.9 (0.8–1.1)	1.3 (1.1–1.6)
Primary	14.6	26.1 (24.1–28.2)	31.5 (29.4–33.6)	1.3 (1.1–1.6)	1.7 (1.4–2.1)
Pre-primary	38.5	47.7 (46.2–49.3)	35.3 (34.0–36.6)	3.4 (2.9–3.9)	2.0 (1.7–2.3)
Total	100.0	31.7 (30.8–32.6)	30.8 (30.0–31.7)	-	-
RII^a^	-	8.4 (7.0–10.0)	2.4 (2.0–2.8)	-	-
SII (%)^a^	-	43.4 (40.2–46.7)	16.3 (13.3–19.3)	-	-
White Women					
Tertiary	19.6	21.6 (18.9–24.3)	22.7 (19.9–25.0)	1.0	1.0
Secondary	34.9	21.3 (19.2–23.4)	26.8 (24.6–28.8)	1.0 (0.8–1.2)	1.3 (1.1–1.7)
Primary	13.1	28.7 (25.2–32.2)	30.5 (27.2–33.7)	1.5 (1.2–1.8)	1.7 (1.3–2.2)
Pre-primary	32.5	51.2 (48.6–53.8)	35.3 (33.3–37.2)	3.8 (3.2–4.6)	2.0 (1.6–2.5)
Total	100.0	32.0 (30.7–33.4)	29.5 (28.4–30.7)	-	-
RII^a^	-	8.4 (6.5–11.0)	2.5 (2.0–3.3)	-	-
SII (%)^a^	-	44.1 (39.2–49.0)	18.1 (13.5–22.8)	-	-
Brown Women					
Tertiary	8.4	17.7 (14.1–21.3)	21.9 (17.9–25.9)	1.0	1.0
Secondary	31.3	17.3 (15.5–19.2)	27.4 (25.2–29.6)	1.0 (0.7–1.3)	1.4 (1.0–2.0)
Primary	16.0	23.5 (20.6–26.4)	31.9 (28.8–35.0)	1.4 (1.1–1.9)	1.9 (1.3–2.6)
Pre-primary	44.2	44.4 (42.3–46.4)	35.3 (33.5–37.1)	3.7 (2.9–4.8)	2.1 (1.6–2.8)
Total	100.0	30.3 (29.0–31.6)	31.8 (30.6–32.9)	-	-
RII^a^	-	10.0 (7.8–12.8)	2.3 (1.8–2.9)	-	-
SII (%)^a^	-	45.1 (40.6–49.6)	14.5 (10.2–18.8)	-	-
Black Women					
Tertiary	8.1	33.8 (21.9–45.6)	32.8 (24.6–41.0)	1.0	1.0
Secondary	30.8	23.2 (18.0–28.5)	32.6 (27.4–37.8)	0.6 (0.3–1.1)	1.0 (0.6–1.7)
Primary	16.3	28.6 (22.2–34.9)	36.1 (30.0–42.1)	0.8 (0.4–1.5)	1.2 (0.7–2.2)
Pre-primary	44.7	49.6 (45.3–53.8)	35.6 (32.2–39.0)	1.9 (1.1–3.4)	1.0 (0.6–1.7)
Total	100.0	36.7 (34.0–39.4)	34.5 (32.1–37.0)	-	-
RII^a^	-	5.7 (3.1–10.4)	1.0 (0.6–1.8)	-	-
SII (%)^a^	-	38.9 (26.6–51.2)	1.5 (−9.2–12.2)	-	-

*RII* relative index of inequality; *SII* slope index of inequality; *OR* odds ratio; *CI* confidence interval

^a^The RII, SII and OR were adjusted for age as a discrete variable (years)

^b^The prevalence of hypertension was adjusted for age groups (Table 1) by means of marginal modeling and interactions with the sociodemographic factors. The standard population was the total PNS population (men and women)

NOTE: All estimates are based on the sample of 59,402 adults aged 18 years or older, considering the appropriate sampling weights. Hypertension was defined as systolic blood pressure ≥ 140 mmHg and/or diastolic blood pressure ≥ 90 mmHg, and/or self-reported use of antihypertensive medications in the last 2 weeks (proportion of missing data of 1.0 %)

After adjustment for age, the prevalence of hypertension was higher among women with the lowest level of education, and among women who self-identified as brown or black. On the other hand, the prevalence of hypertension was similar among men across education strata, and there were no statistically significant differences between racial subgroups.

We found no statistically significant association between men with the lowest education and odds of hypertension occurrence (OR = 1.1; 95 % CI 0.9–1.2). In contrast, women with the lowest education level presented nearly two-fold increased odds of hypertension. As for the odds of having hypertension in intersections with gender and race, the association with the lowest education was 2.0 (95 % CI 1.6–2.5) for white women, and 2.1 (95 % CI 1.6–2.8) for brown women; no statistically significant association was observed for black women and among men across racial strata.

**Fig. 1:**
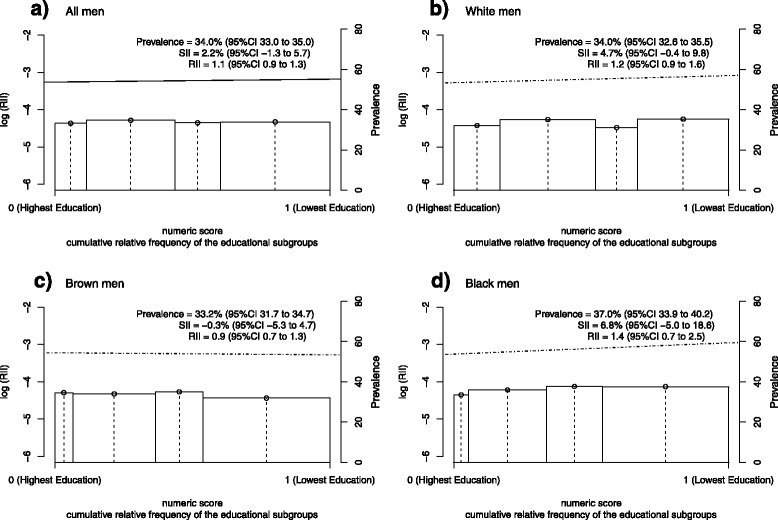
Logarithm of RII and prevalence of hypertension adjusted for age among men in Brazil, 2013. The x-axis denotes the cumulative relative frequency of the study population according to education levels. The numeric score was calculated from the median values of the cumulative relative frequency corresponding to each educational category - indicated by the vertical dotted lines ᅟ

**Fig. 2:**
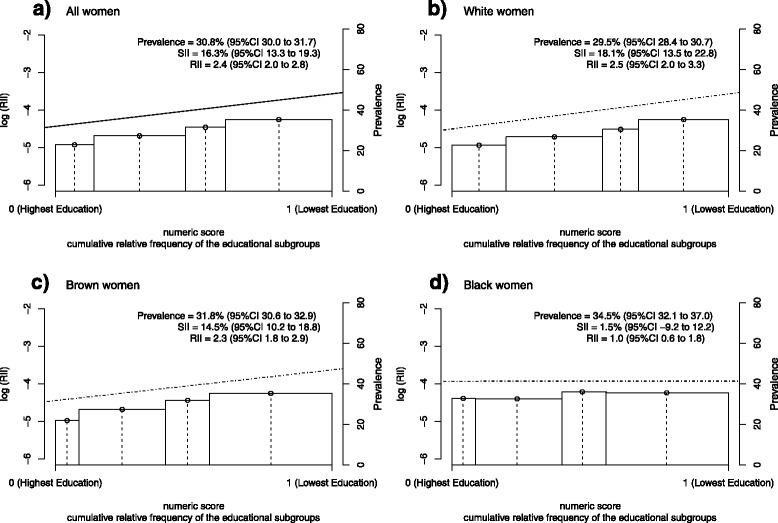
Logarithm of RII and prevalence of hypertension adjusted for age among women in Brazil, 2013. The x-axis denotes the cumulative relative frequency of the study population according to education levels. The numeric score was calculated from the median values of the cumulative relative frequency corresponding to each educational category - indicated by the vertical dotted lines

The age-adjusted Relative Index of Inequality (RII) and the Slope Index of Inequality (SII) summarized the educational inequalities related to the prevalence of hypertension. The relative and absolute sizes of the inequalities for men were respectively 1.1 (95 % CI 0.9 to 1.3) and 2.2 % (95 % CI −1.3 to 5.7), indicating no linear relationship between education and hypertension occurrence (Fig. 1). The RII and SII were, respectively, 2.4 (95 % CI 2.0 to 2.8) and 16.3 % (95 % CI 13.3 to 19.3) for women, which denote a strong and monotonous association in an inverse direction (Fig. 2). In particular, the age-adjusted RII and SII were approximately 1.0 or 0.0 among black women, since there was a homogeneous distribution in the prevalence of hypertension across educational subgroups.

## Discussion

The PNS was the first nationwide survey on blood pressure levels among Brazilian adults aged 18 years or older. To our knowledge, these are the first analyses of socioeconomic inequalities in the occurrence of hypertension including a joint stratification of the study population by gender and race. Further, this is one of the few studies assessing a socioeconomic gradient in hypertension among adults living in a middle-income country. In addition, we utilized the Relative Index of Inequality (RII) and the Slope Index of Inequality (SII), two summary measures still underexplored in epidemiologic research, which properties would help benchmarking and comparing health inequalities.

The age-adjusted prevalence of hypertension was 34.0 % and 30.8 % among men and women, respectively. Black and brown women had a higher prevalence than whites, whereas no racial differences were observed among men. White and brown, but not black women, showed a graded inverse association between hypertension and educational attainment; while among men, there was non-statistically significant association in all racial subgroups. Finally, the RII and the SII, summarizing the relative and the absolute sizes of educational inequalities in hypertension across sociodemographic strata of interest, provided easily interpretable estimates, i.e. an inverse gradient among white (RII = 2.5, SII = 18.1 %) and brown women (RII = 2.3, SII = 14.5 %), and a homogeneous distribution of the hypertension prevalence in multiple educational subgroups among black women and among men.

The higher prevalence of hypertension presented among men is consistent with the general pattern observed in middle- and high-income regions and countries [3, 14, 15]. For example, prevalence of hypertension in Argentina (1988–2013) [29] was 34.5 % among men and 29.0 % among women, which was similar to findings from Cuba 2010–11 (34.1 % vs. 27.9 %) [30] and China 2009–10 (31.2 % vs. 28.0 %) [31]; also, men have a higher occurrence of this condition in Switzerland 1999–2009 (40.5 % vs. 28.3 %) [32] and England 2006 (32.9 % vs. 27.3 %) [33]. It is noteworthy that the excess risk among Brazilian men compared to women occurred especially across those self-identified as white or brown, with more than secondary education level.

It is widely accepted that black individuals have a higher risk of hypertension [34–36]; data from Cuba [30], however, show that such pattern is not universal. In our multi-stratified analysis, this relationship was restricted to black women. The fact that such singularities exist across gender and race strata are even more evident when educational attainment is considered; the excess burden of hypertension endured by black women compared to whites increased with education level. These findings are consistent with the evidence of racial disparity in hypertension, independent of socioeconomic or behavioral factors, mainly among women [7, 37]. One line of argument regards to the cumulative effects of social disadvantages, which still include environmental and psychosocial factors, in addition to stressors resulting from interpersonal or institutional racism [8, 38].

In general, hypertension risk is inversely associated with education [15], mostly among women and less consistently among men [9–11, 39]. In the Brazilian PNS data, however, the picture seemed to be more complex. The hypertension-education association was also inverse among white and brown women, but non-statistically significant among blacks; for men, we observed a similar age-adjusted proportion of hypertension across intersections with gender, race and education. Education is one among several dimensions of individual-level socioeconomic position (SEP) and tends to influence and correlate with other SEP markers [40]. Thus, low educational attainment may directly or indirectly influence risk factors for hypertension through several mechanisms, such as poor diet due to lack of information, access or financial resources, and psychosocial stress due to hazardous occupations or perceived discrimination, among others [15, 40]. In this aspect, our findings suggest that the construct validity of education may vary in intersections with gender and race. Here, the association between education and hypertension was modified by multiple sociodemographic factors, i.e. low education was an important risk factor for hypertension among women compared to men only among individuals self-identified as white or brown – a topic that requires deeper understanding.

Health inequalities are often reported based on a single domain of difference (e.g. gender, race *or* socioeconomic position). Intercategorical approaches allow for comparability of a greater number of social identities and positions, in order to explicit the burden of hypertension among those at the different sociodemographic intersections [41]. This multidimensional analysis, by the way, highlights the inclusion of the populations in a nexus of social privilege and oppression simultaneously (e.g. white low-education men; black high-education women), as well as acknowledges the interplay of different axes of exclusion and marginalization [42]. Therefore, the validity of health inequality research can benefit from an intersectionality theoretical framework [41, 42], e.g. investigating both heterogeneity of effects and social processes producing health inequalities.

Potential limitations of our study should be mentioned. First, socioeconomic position is a complex construct and a variety of indicators may be utilized, like income, wealth, occupation and other individual and contextual markers and indexes [42–44]. Our analyses were based exclusively on the level of education, with several intrinsic limitations, such as the differential economic and social returns across gender and race strata, over time and geopolitical context, and the lack of information about the quality of education. However, education is widely used in social and epidemiological research because of several attributes: easy measurement; substantial information validity; stability along adult life, and thus less subject to negative adult health selection (reverse causality). Second, like most researchers, we chose racial self-identification as our criterion, but caution in required when comparing results; their social and epidemiologic meaning may vary across historical contexts, and there is no established gold-standard for race measurement. Also, the racial composition of study population may vary according to the classification scheme, and socioeconomic disparities are wider when the race variable is defined by interviewers rather than self-identified [45].

## Conclusion

The findings observed in this representative sample of the Brazilian adult population offer a series of contrasting details to the established social patterning of hypertension, thus highlighting the importance of assessing multiple sociodemographic intersections, e.g. gender-race-education, in health inequalities research. Further analyses should explore jointly stratified associations with degree of awareness, treatment and control of hypertension. Also, associations with blood pressure should be explored separately for systolic, diastolic, and pulse pressure, as well as for ambulatory blood pressure and preclinical indicators, e.g. vascular reactivity and endothelial dysfunction. The use of comprehensive measures, e.g. RII and SII, can provide insights and useful information for monitoring health inequalities in an intersectional perspective.

## Abbreviations

CI: Confidence interval
IBGE: Brazilian Institute of Geography and Statistics
OR: Odds ratio
PDA: Personal digital assistant
PNS: Brazilian National Health Survey
PSU: Primary units of sampling
RII: Relative index of inequality
SEP: Socioeconomic position
SII: Slope index of inequality
